# Pharmacokinetics of fluorobenzyl polyethylene glycol conjugated tetraiodothyroacetic acid (NP751), a novel anticancer thyrointegrin α_v_β_3_ antagonist

**DOI:** 10.3389/fphar.2022.902141

**Published:** 2022-11-28

**Authors:** Kazutoshi Fujioka, Bruce A. Hay, Kavitha Godugu, Shaker A. Mousa

**Affiliations:** Pharmaceutical Research Institute, Albany College of Pharmacy and Health Sciences and Nanopharmaceuticals, LLC, Rensselaer, NY, United States

**Keywords:** fb-PMT, NP751, targeted cancer therapeutic, tetrac, thyrointegrin αvβ3 receptor, LC-MS/MS, pharmacokinetic

## Abstract

We have recently reported on the development of fb-PMT (NP751), a conjugate of the thyroid hormone metabolite tetraiodothyroacetic acid (tetrac) and monodisperse polyethylene glycol 36. It exhibited high affinity for thyrointegrin α_v_β_3_ receptor and potent anti-angiogenic and anticancer activity *in vivo*. The objective of the current study is to determine the pharmacokinetics (PK) of fb-PMT in experimental animals, such as mice, rats, and monkeys. NP751 was quantified using a propylene diamine-modified tetraiodothyroacetic acid (DAT) as an internal standard. The limit of quantification (LOQ) for fb-PMT was 1.5 ng/μL and the recovery efficiency was 93.9% with the developed method. The peak plasma concentration (Cmax) and the area under the curve (AUC) results at different doses in mice, rats and monkeys suggest that pharmacokinetics of NP751 is dose-dependent within the dose ranges administered. Results indicate that NP751 has comparable PK parameters that provides enough exposure as a molecularly tumor targeted molecule in multiple species and is a promising anticancer therapeutic.

## Introduction

Anti-cancer therapy has been focused on chemotherapy and radiotherapy to destroy cancer cells (Chen and Kuo, 2017; Mollaei et al., 2021). However, the majority of anti-cancer agents result in serious side effects for the patient (Zhang et al., 2018), thus development of targeted, effective, and safer anti-cancer therapy is needed (Li et al., 2019). Integrin α_v_β_3_ is a potential target for anti-cancer therapy because it is over-expressed in tumor cells and modulates angiogenesis (Davis et al., 2009; Davis and Mousa, 2017; Debreli Coskun et al., 2020). Thus, antagonists of integrin α_v_β_3_ have great potential as cancer therapeutics (Brooks et al., 1994; Mizejewski, 1999; Cox et al., 2010; Desgrosellier and Cheresh, 2010).

We have previously reported that P-bi-TAT, a polydisperse PEG 4000 conjugated to 2 molecules of the α_v_β_3_ active thyroid hormone metabolite tetraiodothyroacetic acid (tetrac) (Davis et al., 2009; Davis and Mousa, 2017; Debreli Coskun et al., 2020), was effective against several different cancer types, with potent effects on solid tumors, (Rajabi et al., 2019; Karakus et al., 2020) and no adverse effects in mice after 28 days of subcutaneous dosing at 30–100 mg/kg (unpublished data). Although the anti-cancer properties of P-bi-TAT were quite promising, the molecule had synthesis scalability issues.

**FIGURE 1:**
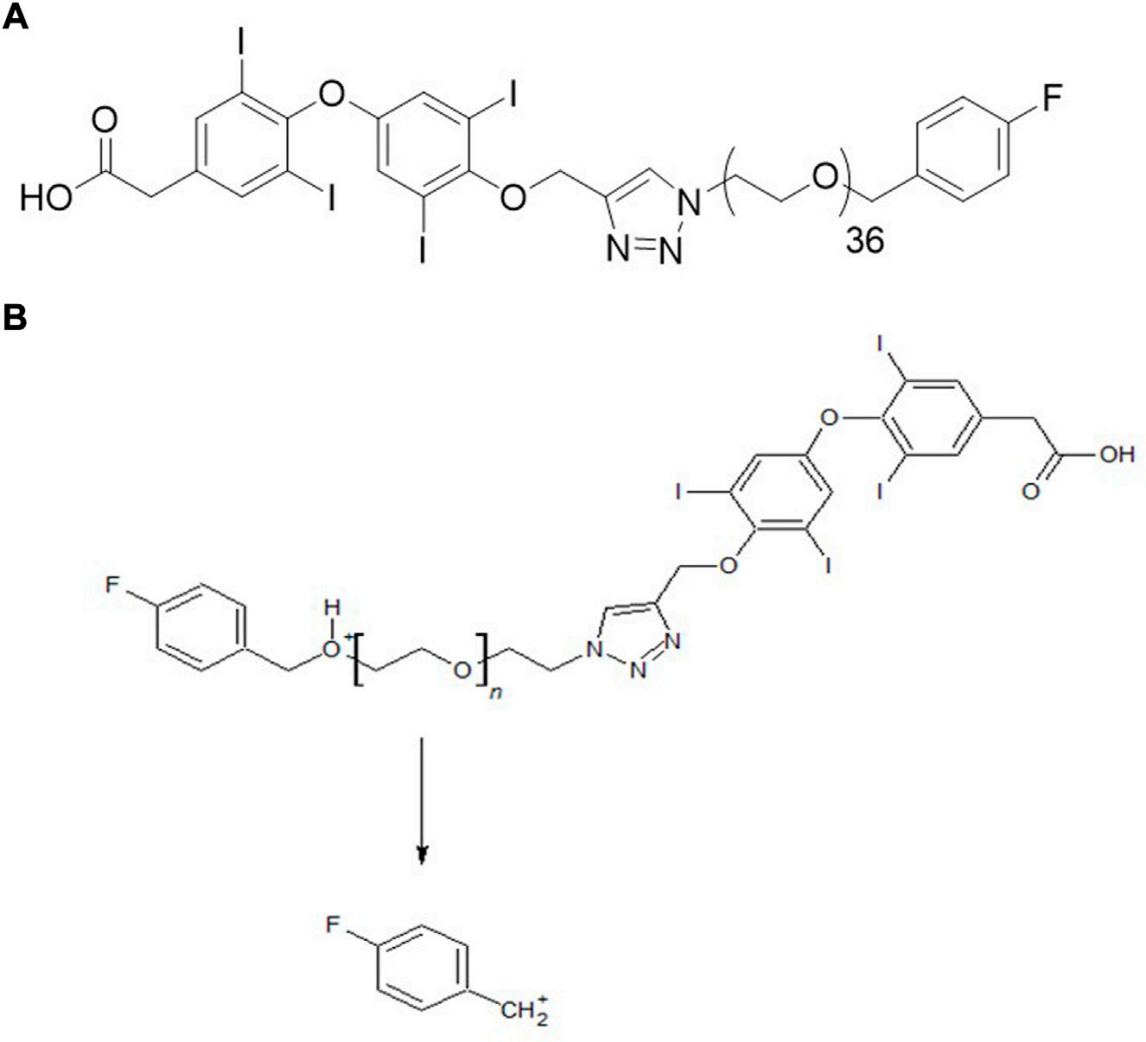
**(A)** Chemical structure of fb-PMT (NP751). **(B)** Plausible MRM transition of fb-PMT (NP751).

We, therefore, developed a new molecule, fb-PMT, a mono-disperse PEG36 conjugated with tetrac, (Figure 1A) with a readily scalable synthesis and a high affinity for the integrin α_v_β_3_ receptor (IC_50_ 0.23 nM) (Hay et al., 2021). Our previous studies showed the anti-cancer effects in several cancer cell lines including Glioblastoma Multiforme, pancreatic and acute myeloid leukemia cancers in mice (Hay et al., 2021). We also observed no toxicity in mice at high dose 100 mg/kg administrated subcutaneously for 28 days (unpublished data). The high anti-cancer efficacy and safety of NP751 prompted us to investigate it is pharmacokinetic (PK) properties in rodents (mice and rats) and monkeys.

The objective of this study was to develop a quantitative LC-MS/MS method for fb-PMT and to evaluate the PK parameters of fb-PMT, such as peak plasma concentration (Cmax) and area under the curve (AUC), in mice, rats and monkeys. This required a quantitative liquid chromatography-tandem mass spectrometry (LC-MS/MS) method for fb-PMT for bioanalytical assays. Although it had a high molecular weight (Mw 2522.9), we were able to develop a sensitive multiple-reaction-monitoring (MRM) method using a pseudo-molecular ion for monodisperse fb-PMT, in contrast to polydisperse P-bi-TAT, which required collision-induced dissociation (CID), of the precursor ion (Fujioka et al., 2021).

## Materials and methods

### Chemicals and biologicals

fb-PMT was synthesized and purified as described earlier at a contract manufacturing organization (Dalton Pharma Services, Toronto, CANADA) under Good Manufacturing Practices (GMP) (Hay et al., 2021). Diamino-propane-tetraiodothyroacetic acid (DAT) was synthesized by formerly DPX Fine Chemicals, Patheon-ThermoFisher (Regensburg, Germany). Acetonitrile, methanol, water, and formic acid were LC-MS grade and purchased from Sigma-Aldrich (St. Louis, MO, United States). Male and female C57BL/6 mice, 4–5 weeks old, weighing 20–22 g and Sprague Dawley Rats, weighing 150–200 g were purchased from Taconic Laboratories (Germantown, NY, United States). Monkey plasma samples were purchased from Bioreclamation IVT (New York, NY).

### fb-PMT (NP751)-treatment in mice

fb-PMT-treatment of mice was carried out in the animal facility of the Veterans Affairs (VA) Medical Center (Albany, NY), and the experimental protocol was approved by the Institutional Animal Care and Use Committee of the VA. Mice were housed under controlled conditions (temperature: 20–24°C; humidity: 60–70%). Mice were allowed to acclimatize for at least 1 week prior to the start of treatments. fb-PMT (1, 3, 6, 10 mg/kg body weight) was administered subcutaneously to 4 mice, and plasma was collected at 0.5, 1, 2, 3, 4, 5, 6, 8, 10, 12, 16, 20 and 24 h. Plasma samples were stored at −80°C for further analysis.

### fb-PMT-treatment in rats

The rat study was conducted by Inotiv (Lafayette, IN United States). Briefly, fb-PMT (5, 15, 30 mg/kg body weight) was administered subcutaneously to male and female Sprague Dawley rats (n = 3 each) daily for 14 days. Samples were collected at the following target timepoints on Day 1: pre-dose, 0.5, 1, 3, 6, 9, 12 and 24 h following the completion of dosing. Samples were collected at the following target timepoints on Day 28: pre-dose, 0.5, 1, 3, 6, 9, 12 and 24 h following the completion of dosing. Plasma samples were stored at -80°C for further analysis.

### fb-PMT-treatment in monkeys

The monkey study was conducted by a contract research organization. Briefly, fb-PMT (2.5, 7.5, 15 mg/kg body weight) was administered subcutaneously to male and female cynomolgus monkeys (n = 2 each) daily for 28 days. Samples were collected at the following target timepoints on Day 1: pre-dose, 0.5, 1, 3, 6, 9, 12 and 24 h following the completion of dosing. Samples were collected at the following target timepoints on Day 28: pre-dose, 0.5, 1, 3, 6, 9, 12 and 24 h following the completion of dosing. Plasma samples were stored at -80 °C for further analysis.

### Preparation of plasma samples with liquid-liquid extraction (LLE)

50 μL of mouse, rat, or monkey plasma with or without fb-PMT and an internal standard (IS) of DAT (150 ng/ml) were vortex-mixed with 1 ml of acetonitrile/water (80/20 v/v) for 30 min. After centrifugation, solvent was evaporated under a nitrogen stream at 50°C. Extracts were reconstituted with 50 μL of acetonitrile/water (80/20 v/v).

### LC-MS/MS instrumentation

An API-4000 mass spectrometer (Sciex, Framingham, MA) equipped with Shimadzu UPLC system (Kyoto, Japan) was used for LC-MS/MS analyses. A Kinetex 2.6 μm Biphenyl 100 LS column (50 × 2.1 mm, Phenomenex, Torrance, CA) was used for reversed-phase separation. Mobile phases were (A) water containing 0.1% formic acid and 5% acetonitrile and (B) acetonitrile with 0.1% formic acid. The flow rate was 0.4 ml/min, and the gradient was linear from 25% B to 95% B for 2–4 min. The oven temperature was 40°C and the injection volume was 5 μL. Electro-spray ionization (ESI) was used in positive MRM mode. Mass transitions for analytes were Q1/Q3: 841.991/109.26 (fb-PMT), 862.9/845.7 (IS). The operative parameters of the mass spectrometer were as follows: declustering potentials (DP): 56 V; entrance potentials (EP): 10; collision energies (CE): 90 eV; collision cell exit potential (CXP), 6 V; curtain gas (CUR), 10 psi; gas 1 (GS1, nebulizer gas) 40 psi; gas 2 (GS2, heater gas) 40 psi; ion spray voltage (IS), 5500 V; temperature (TEM), 500 °C; collision activate dissociation (CAD) gas: 9 psi; dwell time: 150 ms. Nitrogen was used for the curtain, source, and exhaust gases. A standard curve was obtained using standard solutions of fb-PMT with concentrations of 3000, 1000, 300, 100, 30, 10, 3, and 1 ng/ml.

### Determination of recovery efficiency

Recovery efficiency was calculated with the response of extracts from spiked plasma sample (sample) and blank plasma sample (blank) with spike after extract as follows:

Recovery efficiency (%) = Response in sample/Response in blank * 100

### Determination of limit of quantification (LOQ) and limit of detection (LOD)

The limit of quantification (LOQ) and the limit of detection (LOD) was determined using data of six injections of a standard solution (9.8 ng/ml). LOQ and LOD were calculated using the standard deviations (SD) of signals for the analyte and the slope of linear regression curve as follows:

LOQ = 10 x SD/slope
LOD = 3 x SD/slope

### Statistical analysis

It was performed with GraphPad Prism 7 software. Data are presented as mean ± SD. For comparison between groups, ANOVA was used. *p < 0.05 were considered statistically significant.

## Results

### Development of bioanalytical method for fb-PMT

The ionization of fb-PMT was studied in positive and negative modes. A deprotonated pseudo-molecular ion [M-H]^-^ of fb-PMT was detected in negative mode (data not shown). The MW of fb-PMT calculated with the *m/z* of the deprotonated pseudo-molecular ion was consistent with the theoretical values. While observing the molecular ion was useful for confirming molecular weight, the intensity of the deprotonated molecular ion [M-H]^-^ was very weak, so it was not applicable to MRM.

In the positive ion mode, a triply charged ion of fb-PMT at m/z 842.0 was detected, which was much stronger than singly or doubly charged ions (Supplementary Figure S1). The MRM method was optimized using the strongest ion as a precursor ion and a fluorobenzyl cation as a product ion (Figure 1B). Under the LC-MS/MS conditions, the retention time for fb-PMT was 3.8 min. The LC-MS/MS method for fb-PMT standard solutions with IS was linear (r = 0.9923) from a concentration of 9.8–2500 ng/ml (Supplementary Figure S2). The LOQ for fb-PMT was estimated to be 8.8 ng/ml under the current conditions. Accuracy was 100.4%; and recovery was 75.1%. The linearity, recovery, accuracy and LOQ were sufficient for the PK study.

**FIGURE 2:**
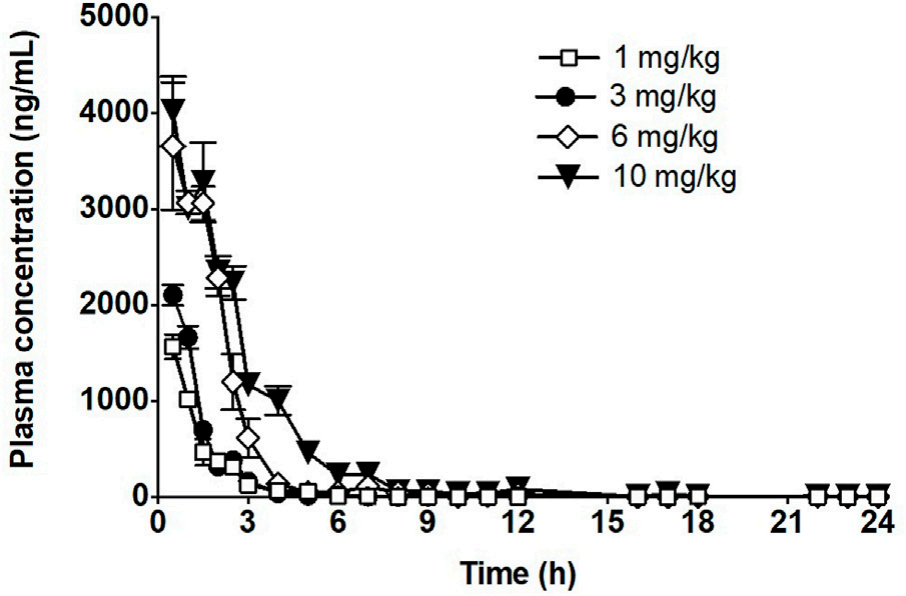
Time course of fb-PMT in mouse plasma following subcutaneous administration of fb-PMT. Data represent mean levels ±SD, (Hay et al., 2021).

The time course of fb-PMT concentrations in mouse plasma is shown in Figure 2. fb-PMT plasma Cmax and AUC dose relationship is shown in Supplementary Figure S3. The PK and TK parameters in mice are shown in Table 1.

**TABLE 1:** PK parameters of fb-PMT in mice.

Dose (mg/kg)	Cmax (ng/ml)	Tmax (h)	AUC (ng/mL*h)	T_1/2_ (h)
1	1565	1	2118	0.8
3	2103	1	2816	0.8
6	3655	1	7631	1.3
10	4015	1	10677	1.5

**FIGURE 3:**
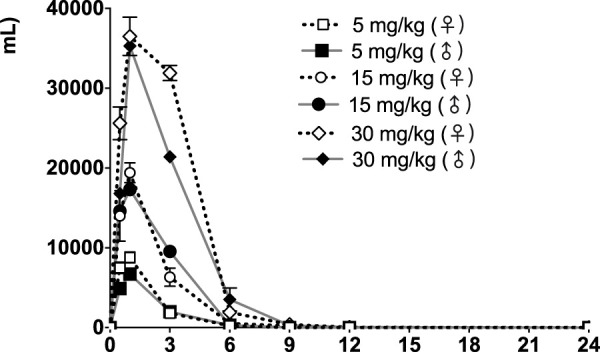
fb-PMT concentration (ng/ml) in the plasma versus time following subcutaneous administration of fb-PMT in male and female Sprague Dawley rats on Day 1. Low dose 5 mg/kg; Medium dose 15 mg/kg; High dose 30 mg/kg. Data represent mean levels ± SD.

**FIGURE 4:**
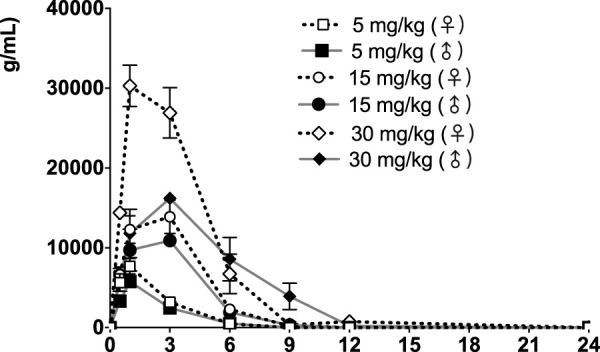
fb-PMT concentration (ng/ml) in the plasma versus time following subcutaneous administration of fb-PMT in male and female Sprague Dawley rats on Day 28. Low dose 5 mg/kg; Medium dose 15 mg/kg; High dose 30 mg/kg. Data represent mean levels ± SD.

The time courses of fb-PMT concentrations in rat plasma are shown in Figure 3 (Day1) and Figure 4 (Day28). The fb-PMT plasma Cmax and AUC dose relationship is shown in Supplementary Figure S4. The PK (Day1) and TK (Day28) parameters in rats are shown in Table 2.

**TABLE 2:** Pharmacokinetic (Day 1) and toxicokinetic (Day 28) parameters of fb-PMT in male and female Sprague Dawley rats.

Day	Dose level (mg/kg)	Sex	C_max_ (ng/ml)	T_max_ (hr)	AUC_last_ (hr*ng/mL)	t_1/2_ (hr)	RA* C_max_	RA* AUC_last_
1	5	Female	8800	1	20000	1.01	-	-
1	5	Male	6710	1	16700	0.868	-	-
1	15	Female	19400	1	49300	1.13	-	-
1	15	Male	17300	1	53800	1.07	-	-
1	30	Female	36500	1	145000	1.15	-	-
1	30	Male	35300	1	117000	1.08	-	-
28	5	Female	7700	1	22000	1.03	0.875	1.1
28	5	Male	5770	1	16600	0.918	0.86	0.994
28	15	Female	13900	3	61000	0.974	0.72	1.24
28	15	Male	10900	3	49400	1.01	0.627	0.918
28	30	Female	30300	1	139000	1.59	0.831	0.959
28	30	Male	16200	3	97300	1.65	0.458	0.828

*RA, Ratio of accumulation as measured by AUC_last_ and C_max_.

**FIGURE 5:**
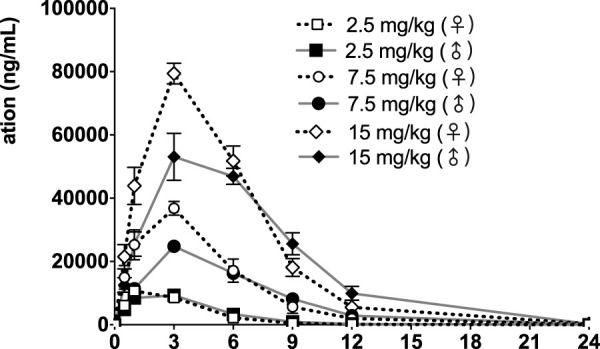
fb-PMT concentration (ng/ml) in the plasma versus time following subcutaneous administration of fb-PMT in male and female monkeys on Day 1. Low dose 2.5 mg/kg; Medium dose 7.5 mg/kg; High dose 15 mg/kg. Data represent mean levels ± SD.

**FIGURE 6:**
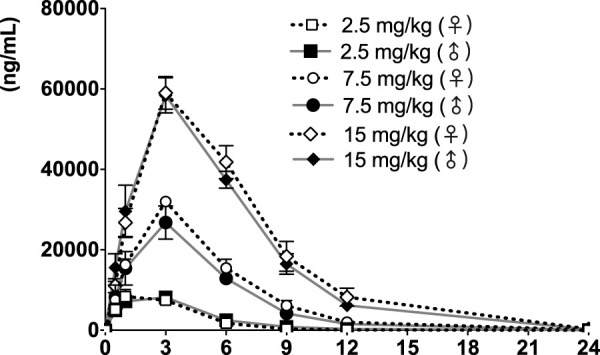
fb-PMT concentration (ng/ml) in the plasma versus time following subcutaneous administration of fb-PMT in male and female monkeys on Day 28. Low dose 2.5 mg/kg; Medium dose 7.5 mg/kg; High dose 15 mg/kg. Data represent mean levels ±SD.

The time course of fb-PMT concentrations in monkey plasma is shown in Figures 5, 6. The fb-PMT plasma Cmax and AUC dose relationship is shown in Supplementary Figure S5. The PK and TK parameters in monkeys are shown in Table 3.

**TABLE 3:** Pharmacokinetic (Day 1) and Toxicokinetic (Day 28) parameters of fb-PMT Following Single and Multiple SC Doses of fb-PMT in Female and Male Cynomolgus Monkeys.

Day	Group	Dose (mg/kg)	Sex	Tmax (h)	Cmax (ng/ml)	T_last_ (hr)	AUC_last_ (hr*ng/mL)	RA* Cmax	RA* AUC_last_	T1/2 (h)
1	2	2.5	Female	1	10700	24	47500	NA	NA	3.07
1	2	2.5	Male	2.3	9340	24	51200	NA	NA	2.68
1	3	7.5	Female	2.3	38100	24	215000	NA	NA	2.68
1	3	7.5	Male	3	24800	24	175000	NA	NA	2.37
1	4	15	Female	3	79400	24	517000	NA	NA	2.56
1	4	15	Male	4.2	54900	24	465000	NA	NA	2.28
28	2	2.5	Female	2.3	9420	24	39200	0.887	0.821	3.48
28	2	2.5	Male	2.3	8250	24	44700	0.891	0.881	3.34
28	3	7.5	Female	3	32000	24	185000	0.841	0.862	2.62
28	3	7.5	Male	3	26800	24	154000	1.06	0.876	2.86
28	4	15	Female	3	59000	24	431000	0.744	0.841	2.6
28	4	15	Male	3	58400	24	401000	1.08	0.865	2.58

*RA, Ratio of accumulation as measured by AUC_last_ and Cmax.

## Discussion

### Development of bioanalytical method for fb-PMT

fb-PMT has both hydrophilic and lipophilic moieties and high affinity for the integrin αvβ3 receptor, resulting in favorable pharmaceutical properties as an anticancer therapeutic. Although its molecular weight is less than 3000, which is the maximum range of typical MSD, it shows predominantly multiple-charged ions in the positive mode. The result is consistent with the chemical properties of PEG conjugates, which tend to coordinate with cations in aqueous solutions. As expected, a fluorobenzyl cation exhibited the strongest product ion; we used this to develop the sensitive MRM method for fb-PMT. The LOQ and recovery of the bioanalytical method was satisfactory under those conditions. A variety of polydisperse PEGylated drug conjugates have been reported; however, PK studies have not been reported, most likely because of their poor sensitivity with traditional LC-MS/MS methods due to their polydispersity (Yin et al., 2020). Yin et al. used LC-TOFMS coupled with pre-column derivatization and MS^ALL^ technique for PEGylated gemcitabine (Mw 70–76 kDa) and conducted its PK study in rats (Yin et al., 2020). The monodisperse fb-PMT conjugate is more readily analyzed in a conventional LC-MS/MS system.

### PK of fb-PMT in mice

Following SC administration, fb-PMT was rapidly distributed into systemic circulation resulting in quantifiable plasma concentrations at 1 h post-dose in all measured mice, and generally remained quantifiable through 12 h. Mean time to peak concentration (Tmax) occurred 1 h post-dose at all four doses tested. Following Tmax, plasma fb-PMT concentrations decreased in a multiphasic manner with mean T_1/2_ values ranging 0.8–1.5 h across all dose levels. Across the dose range (1–10 mg/kg), mean peak (Cmax) exposures to fb-PMT and mean total (AUC) exposures increased in a dose related manner (R^2^ = 0.908 and 0.965, respectively).

### PK of fb-PMT in rats

Following daily SC administration for 28 days, fb-PMT was rapidly distributed into systemic circulation resulting in quantifiable plasma concentrations at 0.5 h post-dose in all measured rats on both Day 1 and Day 28, and generally remained quantifiable through 12 h. Predose fb-PMT concentrations were not observed at all dose levels on Day 1, and at the 5 and 15 mg/kg dose levels on Day 28. There were quantifiable pre-dose fb-PMT concentrations at the 30 mg/kg dose level across both sexes on Day 28. Composite mean time to peak concentration (Tmax) occurred 1 h post-dose on Day 1 and 1–3 h post-dose on Day 28. Elevated Day 28 plasma concentrations at the 12 h relative to the 9 h timepoint were observed for the females administered 5 and 30 mg/kg.

Following daily SC administration of fb-PMT for 28 days, female and male fb-PMT plasma concentrations declined in a multiphasic manner with individual composite mean half-life (T_1/2_) values ranging from 0.87 to 1.7 h. Female composite mean peak (Cmax) and total (AUC_last_) exposures to fb-PMT were comparable (within 2-fold) to the male exposures on Day 1 and Day 28. Across the dose range (5–30 mg/kg), composite mean peak (Cmax) and total (AUC_last_) exposures to fb-PMT generally increased in a dose proportional manner on Day 1 and Day 28. Accumulation was not observed based on RA of Cmax and AUC_last_, which ranged from 0.458 to 0.875 and 0.828 to 1.24, respectively.

Tmax was 1 h on Day 1 and ranged 1–3 h on Day 28 at three doses in female and male rats. T_1/2_ ranged 0.868–1.15 h at three doses in female and male rats on Day 1 (Table 2). T_1/2_ at low and mid doses ranged 0.918 to 1.03 on Day 28; and T_1/2_ at the high dose on Day 28 were slightly longer than T_1/2_ on Day 1 by 38 and 53% (Table 2).

### PK of fb-PMT in monkeys

Following daily SC administration of fb-PMT for 28 days, fb-PMT was rapidly distributed into systemic circulation resulting in quantifiable plasma concentrations at 0.5 h post-dose in all monkeys on both Day 1 and Day 28 and remained quantifiable through 24 h. Quantifiable pre-dose fb-PMT concentrations were not observed at all dose levels on Day 1. Quantifiable pre-dose fb-PMT concentrations were observed at all dose levels on Day 28. Individual time to peak concentration (Tmax) occurred 1–6 h post-dose on Day 1 and occurred 1–3 h post-dose on Day 28. Following Tmax, plasma fb-PMT concentrations decreased in a multiphasic manner with mean T_1/2_ values ranging 2.28–3.48 h across all genders and dose levels on Days 1 and 28.

Female mean peak (Cmax) and total (AUC_last_) exposures to fb-PMT were comparable (within 2-fold) to the male exposures on Day 1 and Day 28. Across the dose range (2.5–15 mg/kg), mean peak (Cmax) exposures to fb-PMT increased in a dose proportional manner and mean total (AUC_last_) exposures to fb-PMT generally increased in a dose proportional manner on Day 1 and Day 28. Accumulation was not observed based on Ratio of accumulation (RA) of Cmax and AUC, which ranged from 0.744 to 1.08 and 0.821 to 0.862, respectively (Table 3).

fb-PMT showed favorable PK parameters without accumulation and differences among different genders (Table 3). A previous study showed that fb-PMT was distributed in the brain after 1 h of the treatment in mice (Hay et al., 2021). The result is consistent with our previous results of the biodistribution of P-bi-TAT (Fujioka et al., 2021) and Fleming et al.‘s results of the chemotherapy drug Camptothecin conjugated with PEG3400 in rats (Fleming et al., 2004; Haverstick et al., 2007). The half-lives of fb-PMT are shorter than the half-lives of P-bi-TAT in rats and in monkeys (Fujioka et al., 2021). Meanwhile, the potency of fb-PMT is similar to that of P-bi-TAT (Rajabi et al., 2019). The high potency and a shorter T_1/2_ of fb-PMT compared to P-bi-TAT may result in the same pharmacological effects with less accumulation into off-target tissues (Mollaei et al., 2021).

## Conclusion

A quantitative bioanalytical method for fb-PMT was developed using LC-MS/MS and LLE. The LOQ for fb-PMT was 1.5 ng/μL and the recovery efficiency was 93.9% with the developed method. The bioanalytical method was used in fb-PMT PK studies to generate PK and TK parameters in mice, rats, and monkeys. The results of this study and the previous study of biodistribution of fb-PMT suggest that fb-PMT is a promising candidate as an anticancer drug. Additional studies, including efficacy in multiple cancer models, accumulation in organs, and metabolism and pharmacodynamics are planned. Furthermore, biodistribution and elimination kinetics will be determined in future studies.

Ethical Approval Statement: All animal studies were conducted at the animal facility of the Veteran Affairs (VA) Medical Center, Albany, NY, United States in accordance with current institutional guidelines for humane animal treatment and approved by the VA IACUC (protocol number 545017).

## Data Availability

The original contributions presented in the study are included in the article/Supplementary Materials, further inquiries can be directed to the corresponding author.
