# Supplementary material for: Pharmacokinetics of fluorobenzyl polyethylene glycol conjugated tetraiodothyroacetic acid (NP751), a novel anticancer thyrointegrin αvβ3 antagonist
Source: Front Pharmacol. 2022 Nov 28;13:902141. doi: 10.3389/fphar.2022.902141 (PMC9742531; doi:10.3389/fphar.2022.902141)

*Supplementary Materials*

**Pharmacokinetic of fb-PMT (NP751), a Novel Anticancer Thyrointegrin α_v_β_3_ Antagonist**

Kazutoshi Fujioka, Bruce A. Hay, Kavitha Godugu, Shaker A. Mousa*

Pharmaceutical Research Institute, Albany College of Pharmacy and Health Sciences, 1 Discovery Drive, Rensselaer, NY 12144

***Correspondence:**

Shaker A. Mousa, PhD, MBA, FACC, FACB

ORCID 0000-0002-9294-015X

Professor of Pharmacology, Executive VP and Chairman,

Pharmaceutical Research Institute

Albany College of Pharmacy and Health Sciences

1 Discovery Drive, Rensselaer, NY 12144

Phone: 01 518 694 7397, Fax: 01 518 694 7567

Email: shaker.mousa@acphs.edu

**Figure S1. Mass spectrum of fb-PMT in positive mode**

**Figure S2. Standard curve for fb-PMT**

**Figure S3. Composite mean fb-PMT plasma Cmax (A) and AUC (B) dose relationship following subcutaneous administration of fb-PMT in mice.**


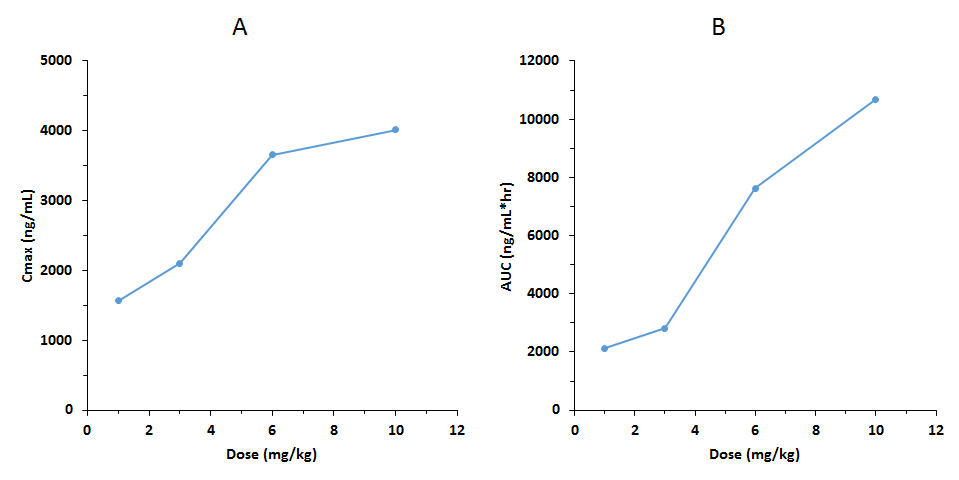


**Figure S4. Composite mean fb-PMT plasma Cmax (A) and AUC (B) dose relationship following subcutaneous administration of fb-PMT in male (M) and female (F) Sprague Dawley rats at different dose levels (5, 15, 30 mg/kg)**


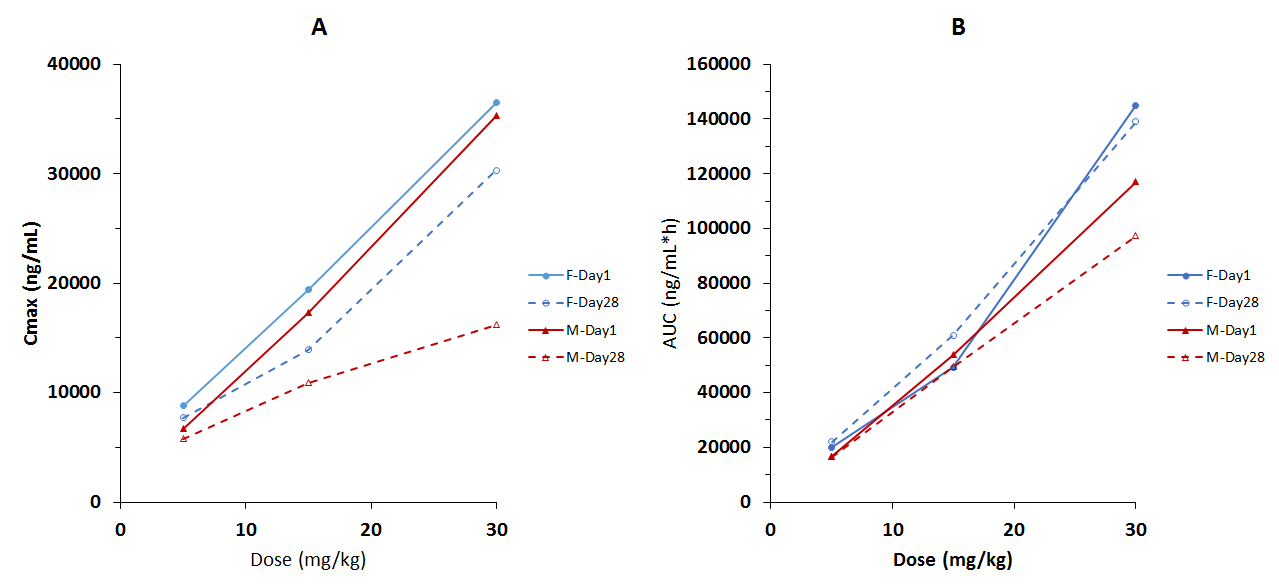


**Figure S5. Mean fb-PMT plasma Cmax (A) and AUC (B) dose relationship following subcutaneous administration of fb-PMT in male and female cynomolgus monkeys.**


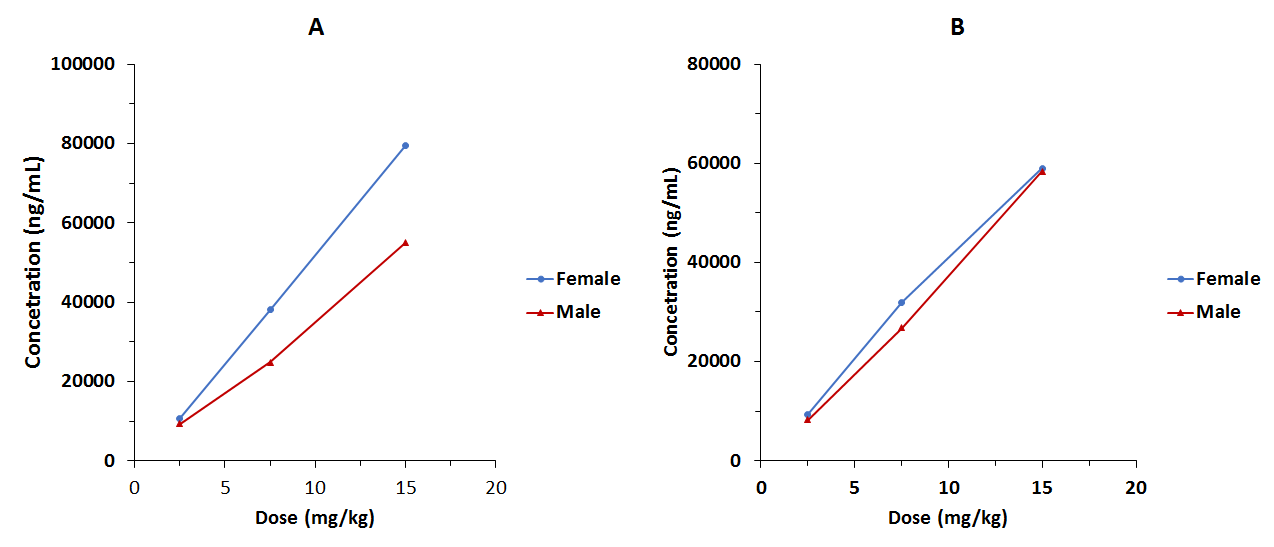

Supplement: Supplementary file 1 [file DataSheet1.docx]
